# *HR4* Gene Is Induced in the *Arabidopsis*-*Trichoderma atroviride* Beneficial Interaction

**DOI:** 10.3390/ijms13079110

**Published:** 2012-07-20

**Authors:** Jorge Sáenz-Mata, Juan Francisco Jiménez-Bremont

**Affiliations:** Division of Molecular Biology, Institute Potosino of Scientific and Technological Research, Camino a la Presa de San José 2055, Col. Lomas 4 sección, C.P. 78216, Apartado Postal 3-74 Tangamanga, San Luis Potosí, San Luis Potosí 78395, Mexico; E-Mail: jorge.saenz@ipicyt.edu.mx

**Keywords:** *Arabidopsis thaliana*, Col-0, Ms-0, *HR4*, *R* genes, *RPW8*, *Trichoderma atroviride*

## Abstract

Plants are constantly exposed to microbes, for this reason they have evolved sophisticated strategies to perceive and identify biotic interactions. Thus, plants have large collections of so-called resistance (R) proteins that recognize specific microbe factors as signals of invasion. One of these proteins is codified by the *Arabidopsis thaliana HR4* gene in the Col-0 ecotype that is homologous to *RPW8* genes present in the Ms-0 ecotype. In this study, we investigated the expression patterns of the *HR4* gene in Arabidopsis seedlings interacting with the beneficial fungus *Trichoderma atroviride*. We observed the induction of the *HR4* gene mainly at 96 hpi when the fungus interaction was established. Furthermore, we found that the *HR4* gene was differentially regulated in interactions with the beneficial bacterium *Pseudomonas fluorescens* and the pathogenic bacterium *P. syringae*. When hormone treatments were applied to *A. thaliana* (Col-0), each hormone treatment induced changes in *HR4* gene expression. On the other hand, the expression of the *RPW8.1* and *RPW8.2* genes of Arabidopsis ecotype Ms-0 in interaction with *T. atroviride* was assessed. Interestingly, these genes are interaction-responsive; in particular, the *RPW8.1* gene shows a very high level of expression in the later stages of interaction. These results indicate that *HR4* and *RPW8* genes could play a role in the establishment of Arabidopsis interactions with beneficial microbes.

## 1. Introduction

Interactions between plants and microbes result in pathogenic or in beneficial associations. It has been revealed that in both interactions a common panel of signaling pathways might participate in the establishment of the equilibrium between plant and microbes or their break-up. Plants appear to detect both pathogenic and symbiotic microbes by a similar set of genes [1]. Beneficial microbes are initially recognized as strange organisms activating the plant’s immune system, which culminates in the establishment of intimate mutualistic relationships. Two strategies have been described to detect microbes, the first involves pattern recognition receptors (PRRs) and the second one disease resistance (R) proteins. Immune signaling in plants is initiated upon perception of non-self molecules or elicitors that are often conserved among different classes of microbes, called pathogen-associated molecular patterns (PAMPs) or microbe-associated molecular patterns (MAMPs) [2]. Plants also respond to endogenous molecules released by microbe invasion, such as cell wall or cuticular fragments called danger-associated molecular patterns (DAMPs) [3–5]. PAMPs, MAMPs or DAMPs are recognized by PRRs. The PRRs are divided into two classes: transmembrane receptor kinases (RKs) and transmembrane receptor-like proteins (RLPs). The stimulation of PRRs leads to MAMP-triggered immunity (MTI) [6,7]. The second strategy of perception involves recognition by plant intracellular receptors (R proteins) of the microbe’s effectors; this recognition induces effector-triggered immunity (ETI), and has led to co-evolutionary dynamics between the plant and the microbe. The effectors are characteristically variable and dispensable in contrast to PAMPs and MAMPs [6,8,9].

The R proteins detect the microbe’s effectors and activate strong defenses. Recently, plant *R* genes have been classified into eight groups based on their amino acid motif organization and their membrane spanning domains. In particular, the Arabidopsis RPW8 proteins are an example of the sixth class of *R* genes, which contains a coiled coil domain (CC), and an *N*-terminal transmembranal domain (TrD) helix that is assumed to localize RPW8 proteins to the endomembrane [8,10]. The two paralogous Arabidopsis Ms-0 *R* genes, *RPW8.1* and *RPW8.2*, confer broad spectrum resistance to multiple powdery mildew isolates that belong to distinct *Erysiphe* spp., and cause diseases in numerous plant species [11]. In contrast, most characterized *R* genes confer resistance to one or a few isolates of a particular pathogen. All tested Arabidopsis accessions contain three homologs of *RPW8*, (*HR1*, *HR2* and *HR3*) that are closely linked to the *RPW8* locus [11,12]. Two basic Arabidopsis haplotypes have been identified at the *RPW8* locus based on the presence or absence of *RPW8.1* and *RPW8.2* genes: One comprises both *RPW8.1* and *RPW8.2* genes and the other is found in susceptible ecotypes that contain the *HR4* gene instead of the *RPW8.1* and *RPW8.2* genes [13,14].

*Trichoderma* species are soil-borne fungi that present a high activity of interaction with soil pathogens and plant roots. *Trichoderma* spp. can reduce the severity of plant diseases by inhibiting plant pathogens in the soil through their highly potent antagonistic activity [15,16]. Moreover, some *Trichoderma* strains can interact directly with roots; as a result, they increase plant growth, resistance to disease and tolerance to abiotic stresses. Like other beneficial microbes, *Trichoderma* elicits Induced Systemic Resistance (ISR) by jasmonic acid/ethylene (JA/ET) dependent pathways and triggers priming responses in the plant. However, the *Trichoderma*-plant cross-talk is dynamic and the expression of defense-related genes of the JA/ET and/or salicylic acid (SA) pathways may overlap, depending on several conditions [17,18]. *Trichoderma* also produces the phytohormones ET and indole-3-acetic acid (IAA), which play roles in interconnecting plant development and defense response [19,20]. Considering the large number of metabolites secreted and the intimate contact with the root epidermis by *Trichoderma*, as well as the expanding list of diverse elicitors produced by fungi, *Trichoderma*’s elicitors may be part of a signaling cascade resulting in greater colonization or plant resistance induction [21].

For a better understanding of plant-microbe interactions, it is essential to study the molecular recognition events. In this sense, we selected the *HR4* gene for this study. Here, we analyzed the gene expression patterns of the *Arabidopsis thaliana HR4* gene under different conditions of interaction with the beneficial fungus *Trichoderma atroviride*. Furthermore, *RPW8.1* and *RPW8.2* genes (from Ms-0 ecotype) paralogs of the *HR4* gene (Col-0 ecotype) were examined during the interaction with *T. atroviride*. Moreover, the analysis of *HR4* gene was extended to bacterial interactions, using beneficial *Pseudomonas fluorescens* and the plant-pathogenic *Pseudomonas syringae* pv. *tomato* DC3000 (Pst DC3000). Finally, *HR4* gene transcriptional profiling in response to hormones such as ET, SA and methyl jasmonate (MeJA) was carried out in order to determine the response to hormones. Despite the fact that the ecotype Col-0 of *A. thaliana* is one of the more studied, the *HR4* gene has not been previously characterized. Thus the data obtained on the *HR4* gene provides valuable information regarding this type of R proteins in Col-0 background.

## 2. Results

### 2.1. The *HR4* Gene (Ecotype Col-0) Expression in Interaction with *Trichoderma*

The *HR4* gene was selected from a cDNA microarray experiment obtained after 48 h of interaction of 25-day-old *A. thaliana* roots with *T. atroviride*. From the results of microarray bioinformatics analysis, we focused on *R* genes that showed a significant induction during this interaction. From ten induced *R* genes, the *HR4* gene showed the highest *Z*-score (5.1) on the microarray experiment.

**Figure 1:**
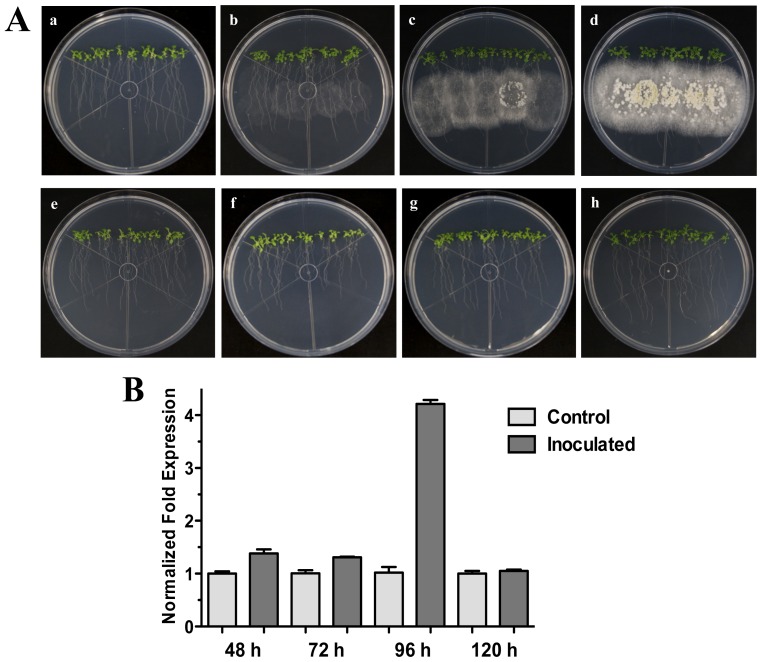
Direct Interaction. Time course development of direct interaction between *Arabidopsis thaliana* plantlets and *Trichoderma atroviride* in MS plates. (**A**) Photographs were taken for 25-day-old Arabidopsis (Col-0) plants inoculated with *T. atroviride* at different time points of interaction: 48 h (**a**), 72 h (**b**), 96 h (**c**) and 120 h (**d**) post-inoculation. Photographs of 25-day-old Arabidopsis (Col-0) control non-inoculated plantlets at different times are indicated: 48 h (**e**), 72 h (**f**), 96 h (**g**) and 120 h (**h**); (**B**) Expression analysis of *HR4* gene in direct interaction. Quantification of *HR4* gene by qRT-PCR, expressed as relative mRNA level compared with control conditions, was calculated after normalization to the Arabidopsis *actin 8* gene using the comparative threshold method. Analyses were performed by triplicate. Control conditions or non-inoculated plantlets (light grey bars) and tester conditions or inoculated plantlets (dark bars) at 48, 72, 96 and 120 hpi, respectively.

qRT-PCR analyses were performed to corroborate the microarray data on *HR4* expression after 48 h of interaction, using 25-day-old plants grown on MS (Figure 1B). An induction of 1.4 times of the *HR4* gene was observed in *A. thaliana* roots after 48 h post-inoculation (hpi) with *Trichoderma* spores, in comparison to control plants without fungus.

Our next step was to analyze additional times of interaction (72, 96 and 120 hpi) when the fungus is more grown and developed (Figure 1A). The results showed that *HR4* highest induction was at 96 hpi, reaching a relative expression of 4.2-fold change (Figure 1B), when the fungus is already well established and beginning to sporulate (Figure 1A).

**Figure 2:**
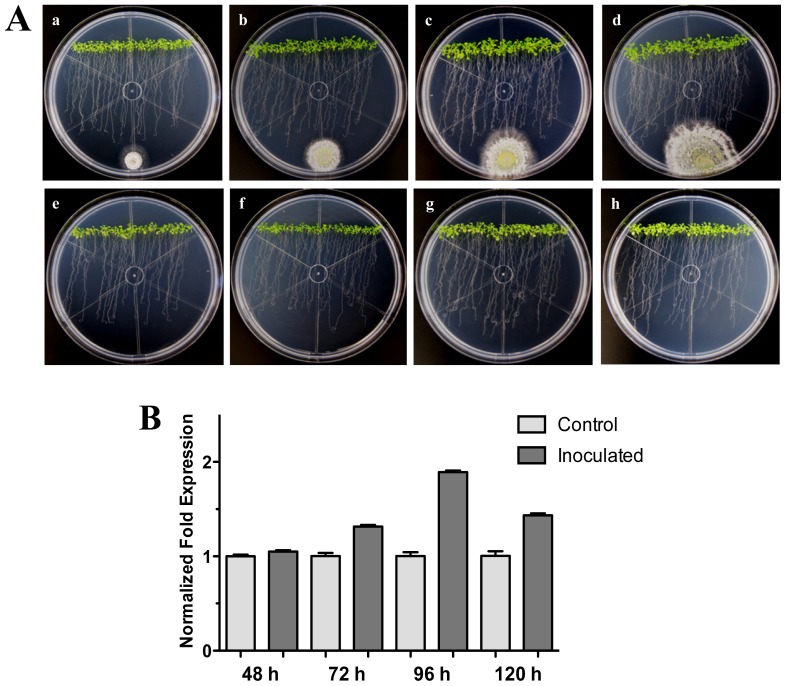
Distant interaction. (**A**) Development of distant interaction of *Arabidopsis thaliana* plantlets with *Trichoderma atroviride*. (**a**), (**b**), (**c)** and (**d**) photographs of 17-day-old Arabidopsis (Col-0) plantlets inoculated with *T. atroviride*, the plantlets were photographed at 48 h (**a**), 72 h (**b**), 96 h (**c**) and 120 h (**d**) post-inoculation. (**e**), (**f**), (**g**) and (**h**) photographs of 17-day-old Arabidopsis (Col-0) control non-inoculated plantlets of 48 h (**e**), 72 h (**f**), 96 h (**g**) and 120 h (**h**); (**B**) Induction of *HR4* gene by *T. atroviride* at a distance. Quantification of *HR4* gene by qRT-PCR, expressed as relative mRNA level compared with control conditions, was calculated after normalization to the Arabidopsis *actin 8* gene using the comparative threshold method. Analyses were performed by triplicate. Control conditions or non-inoculated plantlets (light grey bars) and tester conditions or inoculated plantlets (dark bars) at 48, 72, 96 and 120 hpi, respectively.

We further examined the effect of distance between the *Arabidopsis*-*Trichoderma* interaction. For this, an experiment where *T. atroviride* was inoculated at 3 cm distance from the root tips was carried out. As with the previous interactions, the expression levels for the *HR4* gene were higher at the later stages (96 and 120 hpi) evaluated, when the fungus was more established, and contact was already evident in the Arabidopsis roots (Figure 2A). The maximum expression was at 96 hpi, reaching 1.9-fold in comparison to the control plants (Figure 2B). All these results suggest that the contact of the fungus with the plant roots could activate the recognition system and/or could promote a beneficial interaction establishment in the plant.

**Figure 3:**
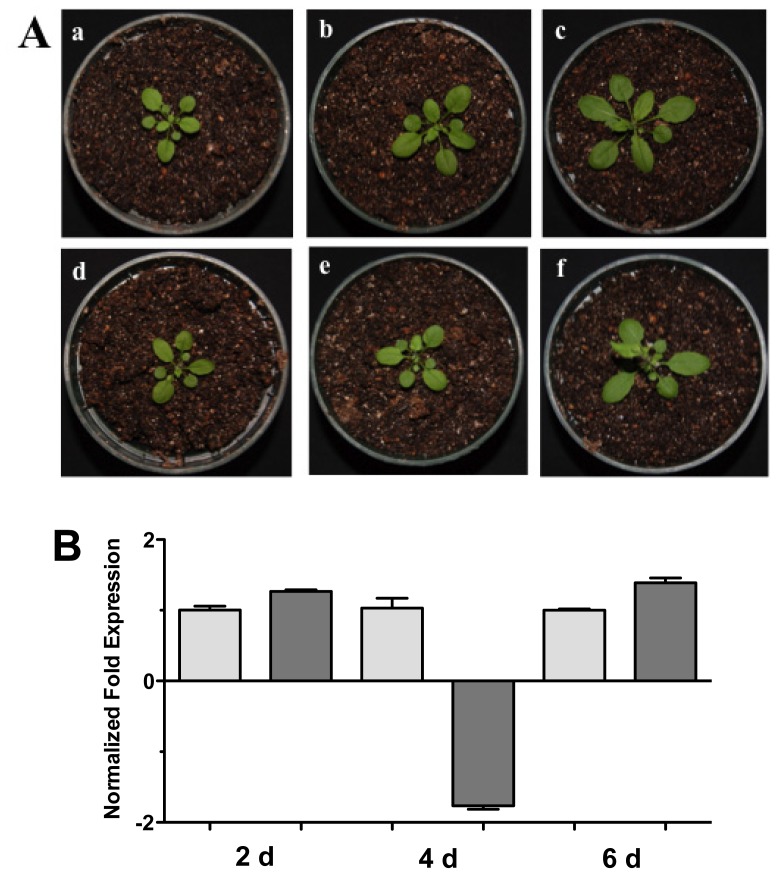
Soil interaction. (**A**) Photographs of Arabidopsis plants inoculated with *Trichoderma* in pots. (**a**), (**b**) and (**c**), 1 month-old Arabidopsis plants in pots inoculated with *Trichoderma atroviride*, the plants were photographed at (**a**) 2 days, (**b**) 4 days, and (**c**) 6 days post-inoculation. (**d**), (**e**) and (**f**), 1 month-old Arabidopsis control non-inoculated plants of (**d**) 2 days, **(e**) 4 days, and (**f**) 6 days; (**B**) Expression analysis of *HR4* gene from Arabidopsis plants grown in soil pots. Quantification of *HR4* gene from aerial part of plants by qRT-PCR, expressed as relative mRNA level compared with control conditions, was calculated after normalization to the Arabidopsis *actin 8* gene using the comparative threshold method. Analyses were performed by triplicate. Control conditions or non-inoculated plants (light grey bars) and tester conditions or inoculated plants (dark bars) at 2, 4, and 6 dpi, respectively.

Complementary to this, we examined the *HR4* expression of the *Arabidopsis-Trichoderma* interaction in soil. Arabidopsis plants in pots with soil were inoculated and the aerial part was used for expression studies at 2, 4, and 6 days post-inoculation (dpi) (Figure 3). Plants inoculated with *T. atroviride* increased aerial parts in comparison to the control plants (Figure 3A). The *HR4* gene expression was measured at these points in time, observing a slight increase in gene transcription at 2 and 6 dpi (Figure 3B).

### 2.2. The *RPW8.1* and *RPW8.2* Genes (Ecotype Ms-0) Expression in Interaction with *Trichoderma atroviride*

**Figure 4:**
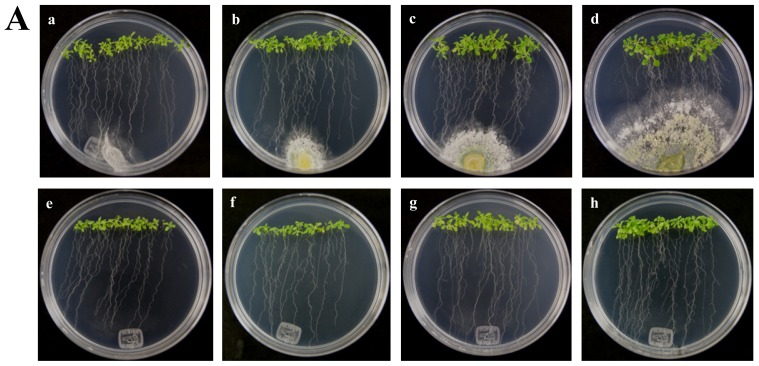

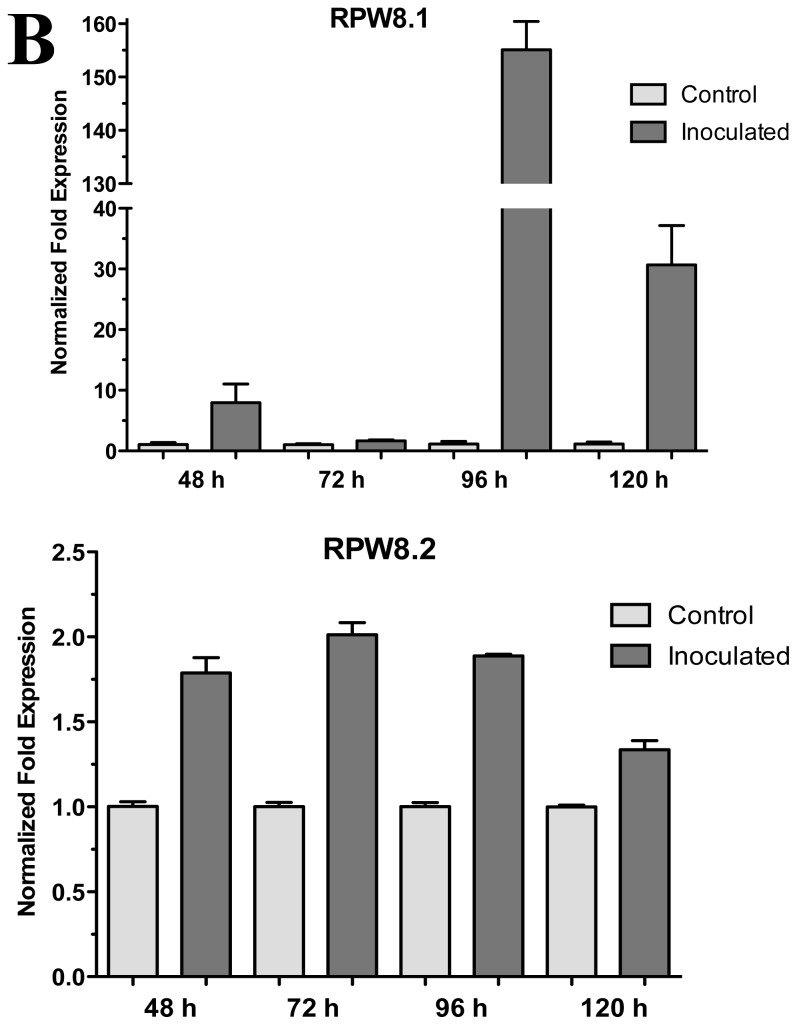
Expression of *RPW8.1* and *RPW8.2* genes from *Arabidopsis thaliana* ecotype Ms-0 in interaction with *Trichoderma atroviride*. (**A**) Development of interaction of *Arabidopsis thaliana* ecotype Ms-0 plantlets with *Trichoderma atroviride*. (**a**), (**b**), (**c**) and (**d**) photographs of 15-day-old Arabidopsis (Ms-0) plantlets inoculated with *T. atroviride*, the plantlets were photographed at (**a**) 48 h, (**b**) 72 h, (**c**) 96 h and (**d**) 120 h post-inoculation. (**e)**, (**f**), (**g**) and (**h**), photographs of 15-day-old Arabidopsis (Ms-0) control non-inoculated plantlets of (**e**) 48 h, (**f**) 72 h, (**g**) 96 h and (**h**) 120 h; (**B**) Induction of *RPW8.1* and *RPW8.2* genes by *T. atroviride* interaction. Quantification of *RPW8.1* and *RPW8.2* genes by qRT-PCR, expressed as relative mRNA level compared with a control conditions, was calculated after normalization to the Arabidopsis *actin 8* gene using the comparative threshold method. Analyses were performed by triplicate. Control conditions or non-inoculated plantlets (light grey bars) and tester conditions or inoculated plantlets (dark bars) at 48, 72, 96 and 120 hpi, respectively.

The *A. thaliana RPW8* locus from ecotype Ms-0 contains two paralogous genes, *RPW8.1* and *RPW8.2*, both of which confer resistance to powdery mildew fungi [14]. These genes have been extensively studied in interactions with pathogenic fungus (Powdery Mildew), but nothing is known about beneficial fungus interaction. According to the above, we used the *A. thaliana* ecotype Ms-0 in interaction with *T. atroviride* for the transcriptional characterization of the *HR4* homologues genes, *RPW8.1* and *RPW8.2*. The *Trichoderma* inoculation was made at the lower end of the Petri dish closed to the plantlets roots, and the gene expression was measure at 48, 72, 96 and 120 hpi (Figure 4A). Both, *RPW8.1* and *RPW8.2* genes were up-regulated, but the maximum expression was observed in the *RPW8.1* gene reaching the highest expression level (155-fold) at 96 hpi, and the lowest expression level (1.67-fold) at 72 hpi. At 48 and 120 hpi the gene expression was 7.9 and 30.6-fold, respectively (Figure 4B *RPW8.1*). With respect to the *RPW8.2* gene, it was up-regulated less than the *RPW8.1*, reaching the induction levels 1.7, 2.0, 1.8 and 1.3-fold at 48, 72, 96 and 120 hpi, respectively (Figure 4B *RPW8.2*). The inoculation with the beneficial fungus resulted in an increase in the lateral roots formation and growth of the aerial part in Arabidopsis seedlings ecotype Ms-0 (Figure 4A), this observation is consistent with that observed with Col-0 ecotype (Figure 2A).

### 2.3. *HR4* Gene Is Induced by Bacterial Interactions

**Figure 5:**
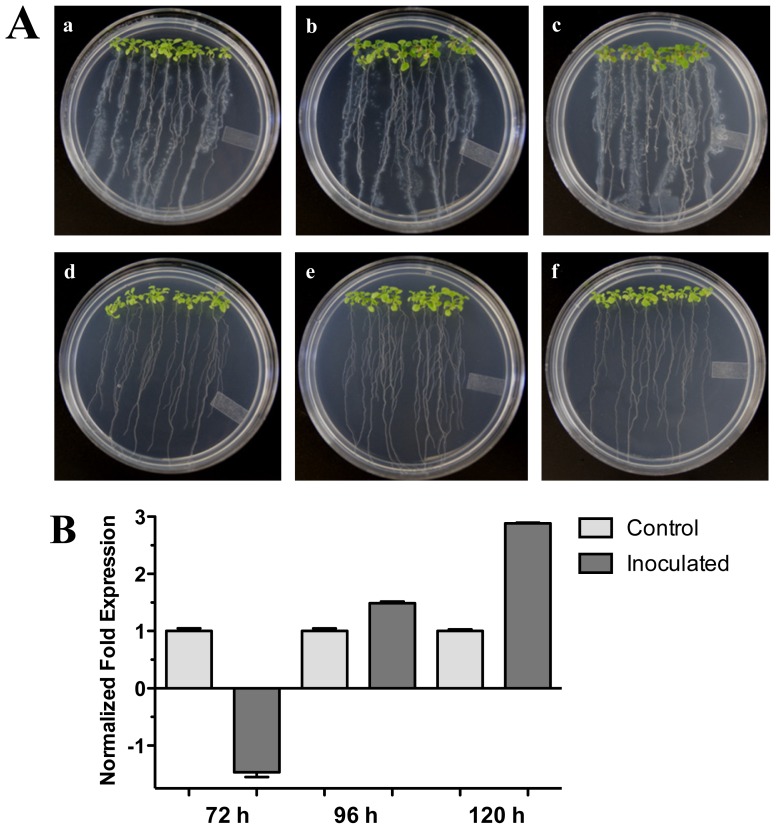
*Pseudomonas fluorescens* interaction. (**A**) Development of the interaction of Arabidopsis with *P. fluorescens*. (**a**), (**b**), and (**c**), photographs of 20-day-old Arabidopsis (Col-0) plantlets inoculated with *P. fluorescens*, the plantlets were photographed at (**a**) 72 h, (**b**) 96 h, and (**c**) 120 h post-inoculation. (**d**), (**e**) and (**f**), photographs of 20-day-old Arabidopsis (Col-0) control non-inoculated plantlets of (**d**) 72 h, (**e**) 96 h and (**f**) 120 h; (**B**) Expression analysis of the *HR4* gene of Arabidopsis in interaction with beneficial bacterium *P. fluorescens*. Quantification of *HR4* gene by qRT-PCR, expressed as relative mRNA level compared with a control condition, was calculated after normalization to the Arabidopsis *actin 8*. Control conditions or non-inoculated plantlets (light grey bars) and tester conditions or inoculated plantlets (dark bars) at 72, 96 and 120 hpi, respectively.

The effect of plant growth-promoting rhizobacterium (PGPR) *Pseudomonas fluorescens* on Arabidopsis (Col-0) 20-day-old seedlings was assessed (Figure 5A). We tested the expression of the Arabidopsis *HR4* gene in interaction with *P. fluorescens* roots inoculated at 72, 96 and 120 hpi. As shown in Figure 5B, a gradual increase of the *HR4* mRNA level with the progress of the interaction was observed, beginning with a repression at 72 h (−1.46), followed by inductions of 1.49 and 2.88-fold at the later stages of interaction.

**Figure 6:**
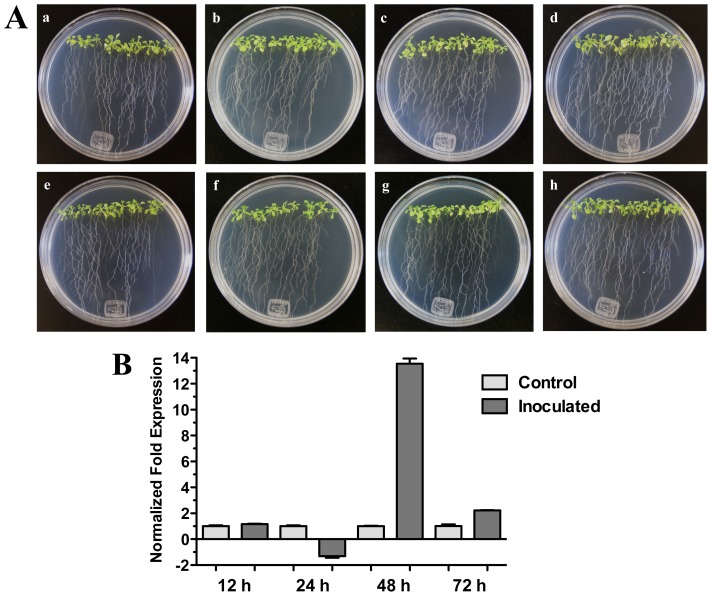
*Pseudomonas syringae* interaction. (**A**) Development of the interaction of Arabidopsis with *P. syringae* pv*. tomato* DC3000. (**a**), (**b**), (**c**) and (**d)** photographs of 20-day-old Arabidopsis (Col-0) plantlets inoculated with *P. syringae*, the plantlets were photographed at 12 h (**a**), 24 h (**b**), 48 h (**c**) and 72 h (**d**) post-inoculation. (**e**), **(f**), **(g**), and (**h**), photographs of 20-day-old Arabidopsis (Col-0) control non-inoculated plantlets of 12 h (**e**), 24 h (**f**), 48 h (**g**) and 72 h (**h**); (**B**) Expression analysis of the *HR4* gene of Arabidopsis in interaction with plant-pathogenic *P. syringae*. Quantification of the *HR4* gene by qRT-PCR, expressed as relative mRNA level compared with a control condition, was calculated after normalization to the Arabidopsis *actin 8*. Control condition or non-inoculated plantlets (light grey bars) and tester conditions or inoculated plantlets (dark bars) at 12, 24, 48 and 72 hpi, respectively.

On the other hand, when Arabidopsis (Col-0) seedlings were inoculated with bacterial pathogenic *Pseudomonas syringae* pv. *tomato* DC3000 a significant increase of *HR4* gene expression was observed at 48 h (13.54-fold) (Figure 6B). In this interaction, bacteria were inoculated in foliar area of 20-day-old seedlings, and initial characteristic symptoms of the *P. syringae* infection were observed at 72 h, with initial chlorosis and anthocyanin production in the center of the rosette seedlings (Figure 6A).

### 2.4. Effect of Phytohormones on the Expression of *HR4* Gene

**Figure 7:**
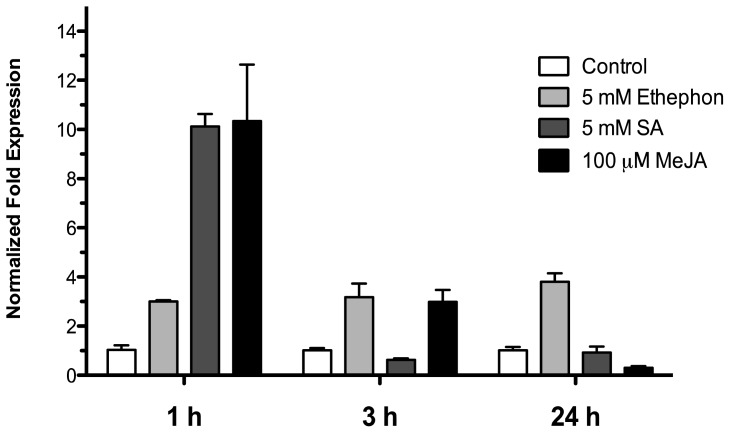
Phytohormonal treatment effects. Expression analysis of the *HR4* gene from Arabidopsis by the addition of phytohormones, 5 mM ethephon, 5 mM SA, and 100 μM MeJA. Control conditions or without phytohormones (white bars) and tester conditions with 5 mM of ethephon (light grey bars), 5 mM SA (dark grey bars), and 100 μM MeJA (black bars) at 1, 3, and 24 h post sprayed, respectively.

To examine the effect of phytohormones involved in biotic stress signaling on the *HR4* gene expression, 15-day-old Arabidopsis (Col-0) seedlings were treated by spraying with 5 mM ethephon, 5 mM salicylic acid and 100 μM methyl jasmonate in a MS solution, control seedlings were treated with an equivalent amount of MS solution. The treated seedlings and controls were harvested at 1, 3, and 24 h after sprayed. As show in Figure 6, with ethephon (ET) treatment the *HR4* gene was induced in early times at 1 and 3 h with 3.00 and 3.18-fold, respectively, and this induction was maintained at 24 h (3.79-fold). For SA and MeJA treatment, we observed a strong initial induction at 1 h of 10.1- and 10.3-fold, respectively. The next times, at 3 and 24 h, the level of expression was down-regulated for the two hormones (Figure 7).

## 3. Discussion

Plants have evolved in constant interaction with beneficial microorganisms, which influence plant growth and development and also plant health. These beneficial microorganisms are indispensable for a sustainable agriculture and environment. Although the plant-pathogen interaction is better understood, the plant-beneficial interaction is less known; hence, it is necessary to understand what is happening at the molecular level between plant and beneficial microorganisms [7]. It is a critical point for providing new strategies to improve plant productivity, while protecting the environment and biodiversity.

Some Trichoderma rhizosphere-competent strains have been shown to have direct effects on plants. These effects are: (i) the promotion of plant growth and development, and thus increased yields [22,23]; (ii) breaking seed dormancy and enhanced rates of seed germination [24–26]; (iii) improved tolerance to abiotic stresses during plant growth, in part due to improved root growth, and enhanced water-holding capacity of plants or enhanced nutrient uptake (phosphorus and several micronutrients) [26–29]; (iv) increased photosynthetic capacity [27]; and (v) systemic induction of plant defenses towards attack by pathogenic microorganisms [22,30]. Several global analyses have been published of the alteration of proteome [31–33] and transcriptome [30,34–36] of plants as a consequence of Trichoderma colonization.

*Arabidopsis thaliana* represents a functional system to study beneficial plant-microbe interactions. In particular, Contreras-Cornejo *et al*. in 2009 [19] reported the beneficial effects of Trichoderma species on plant growth and the development of *A. thaliana*. It has also been reported that the presence of Trichoderma primes the systemic resistance system, a complex signaling mechanism involving diverse hormones pathways. This complex signaling mechanism involving JA/ET-induced systemic resistance (ISR) and/or SA-dependent systemic acquired resistance (SAR) pathways [37]. The activation of ISR and/or SAR in plants is part of the response to microbes by the plants. In order to recognize microbes, plants have a large collection of *R* genes, which directly or indirectly recognize effectors from beneficial and pathogenic fungi or bacteria.

In a microarray of the interaction *A. thaliana* (Col-0)-*T. atroviride* [38] we analyzed the expression of *R* genes, where the *HR4* gene was one of the most strongly induced. For this reason, in this study we focused on *HR4* gene expression during the interaction with this beneficial fungus. At 48 hpi an induction of the *HR4* gene was found; however, at 96 hpi the *HR4* gene showed the highest peak of expression in comparison with the *A. thaliana* seedlings without fungus. The same behavior was observed when the fungus was inoculated at the end of the petri dishes (distance interaction), where the maximum peak of *HR4* gene expression was also at 96 hpi. At this point in time, the fungus development was more evident and the interaction was established in both interactions. When the aerial part of 27-day-old Arabidopsis inoculated with Trichoderma in pots was analyzed transcriptionally, we observed a cyclic behavior of the *HR4* gene with alternated transcript induction (2 and 6 dpi) and repression (4 dpi).

There are only a few reports about the *HR4* gene. Xiao *et al*. [13] reported that the transcription of the *HR4* gene from *A. thaliana* ecotype Col-0 was induced by powdery mildew and *Peronospora parasitica*. In another study, the *HR4* gene induction from an interaction of *A. thaliana* (Col-0) with *Botrytis cinerea* was observed at 12 and 24 hpi [39]. In the present study, we report the first transcriptional regulation of the *HR4* gene in interaction with a beneficial microbe.

The *A. thaliana HR4* gene (ecotype Col-0) is homologue to *RPW8* genes (ecotype Ms-0). The RPW8 locus from accession Ms-0 confers broad-spectrum resistance to powdery mildew. This locus contains two paralogous genes, *RPW8.1* and *RPW8.2*, both of which contribute to resistance [12,13]. The origin of the RPW8 locus is relatively recent, probably after the separation of Arabidopsis from the Brassica lineages, and that *RPW8.1* and *RPW8.2* have evolved from an *HR3*-like predecessor gene by duplication and functional diversification. The *HR4* gene is probably of most recent origin, which appears to have arisen from *RPW8.1* [13,14].

Based on particular differences between Arabidopsis haplotypes (*RPW8* locus and *HR4*), the expression of the *RPW8.1* and *RPW8.2* genes of Arabidopsis ecotype Ms-0 in interaction with *T. atroviride* was assessed. An important finding in the present study was the increased expression of *RPW8.1* and *RPW8.2* genes from Arabidopsis ecotype Ms-0 during the interaction with Trichoderma. The up-regulation of *RPW8.2* gene was maintained in for the first three times with similar levels, which were similar to *HR4* gene levels of ecotype Col-0. Furthermore, the expression profile of *RPW8.1* was like that of *HR4* gene, where at later stages there was an evident increase in mRNA level, especially at 96 hpi with a strong increase in both genes (Figures 1B and 3B). Xiao *et al*. [40] proposed that the increased transcription of RPW8s formed part of an amplification circuit that lead to the accumulation of SA. Therefore, SA formed during the expression of resistance to different pathogens also would induce the accumulation of transcripts of *RPW8.1* and *RPW8.2* via a feedback amplification circuit. Although there is much information about *R* genes in plant-pathogen interactions (e.g., *RPW8* genes), only little is known about *R* genes in beneficial interactions.

In addition to fungal interactions, we analyzed the interaction between *A. thaliana* (Col-0) and bacteria. When the beneficial bacterium, the plant growth promoting rhizobacterium (PGPR) *Pseudomonas fluorescens*, was inoculated into the Arabidopsis roots, an induction of the *HR4* gene specifically in the later stages (120 hpi) was noticed. This result is consistent with that reported by Wang *et al*. [41], who found using microarray analysis that the *HR4* gene was up-regulated in the interaction *A. thaliana*-*P. fluorescens* (FPT9601-T5). On the other hand, in response to the infection by the pathogenic bacterium *P. syringae* pv *tomato* DC3000 on Arabidopsis seedling leaves (Col-0), the *HR4* gene markedly increased the mRNA level at 48 hpi.

Plant hormones are essential for the regulation of plant defense, the importance of salicylic acid (SA), jasmonic acid (JA) and ethylene (ET) as primary signals is well established [42–44]. The interaction of plants with diverse microbes results in changes in the level of these phytohormones, which are positive regulators of defense genes (PRs), transcription factors (WRKYs) and receptors (*R* genes). The *R* genes are the activators of immune response, and simultaneously the *R* gene promoters have *cis*-elements that increase the amplification by feedback positive regulation circuits stimulated by hormonal action [44]. Cross-talk among SA, JA and ET signaling pathways has emerged as an important regulatory mechanism of plant immunity [42,43]. There are different reports about what the hormonal activated pathway in the interaction *Arabidopsis*-*Trichoderma* [20] is. *T. atroviride* induces a delay and overlapping activation of the defense-related genes of the SA and JA/ET pathways against biotrophic and necrotrophic phytopathogens [19,45]. *T. atroviride* P1 and *T. harzianum* T22 are able to induce a long-lasting up-regulation of SA gene markers, after the infection with *Botrytis cinerea* the expression of defense genes increases induced through the JA pathway, culminating in ISR [17], and *T. asperellum* produces a clear ISR through SA signaling cascade [18]. In order to determinate the transcriptional response of the *HR4* gene, expression was measured under hormonal treatment of Arabidopsis seedlings with the exogenous application of SA, MeJA and ethephon phytohormones, which have been implicated in the response to Trichoderma. The *HR4* gene expression was up-regulated with SA and MeJA treatments observing an abrupt induction 1 h post-treatment, and a decrease in expression at later times. On the other hand, the ethephon treatment constantly maintained the up-regulated mRNA level three times. *RPW8.1* and *RPW8.2* genes involved a self-amplification mechanism via the SA-feedback circuit for activation of HR and resistance [40,46]. The response of the *HR4* gene by SA reported in this study, and what was reported for *RPW8.1* and *RPW8.2* genes [40], could be due to *W*-box elements within their regulatory regions.

In the interaction of *T. harzianum* T34 with Arabidopsis plants, a transcriptional regulation of SA-related genes, such as Enhanced Disease Susceptibility 1 (EDS1) and PR-1, was reported [35]. At an early stage (4 h), a strong down-regulation of EDS1 and PR-1 genes was detected, but after 48 h of Arabidopsis root colonization by *T. harzianum* an increase in the expression of these genes was reported [35]. Interestingly, RPW8-mediated resistance is associated with the expression of *PR* genes, and signaling in RPW8-mediated resistance occurs through a feedback amplification loop in the SA pathway, and requires signaling components such as *EDS1*, *EDS5*, *PHYTOALEXIN DEFICIENT 4 (PAD4)*, and *NONEXPRESSOR OF PATHOGENESIS-RELATED PROTEIN 1* (*NPR1*) [11,40,46,47].

The *HR4* gene is classified as an early SA induced gene (early SAIG), as reported by Blanco *et al*. in 2009 [48], who analyzed the early genetic response to SA of Arabidopsis seedlings. Using microarray analysis, they identified 217 genes rapidly induced after 2.5 h SA treatment; 193 by a NPR1-dependent and 24 by a NPR1-independent pathway. The *HR4* gene with a 3.0-fold-change ratio was included in the NPR1-dependent group. In another study by Galon, *et al*. in 2008 [49], they reported the elevate expression of 32 genes related to defense against pathogens in transcriptomic analysis using microarrays from *A. thaliana* and *camta3* (insertion mutants in the Calmodulin binding transcription activators 3 gene) plants, amongst these genes there was the *HR4* gene with a 6.7-fold change ratio, suggesting that CAMTA3 normally suppresses biotic defense responses. In accordance with these findings, our transcription data of *HR4* and *RPW8* genes support the idea that the SA pathway is a key player in the recognition, establishment and/or activation of plant systemic defense by the beneficial fungus *T. atroviride*.

## 4. Experimental Section

### 4.1. Plant Material and Growth Conditions

*Arabidopsis thaliana* (Col-0) seeds were surface-sterilized and sown on Petri dishes containing 0.5× Murashige and Skoog (MS) medium [1.4% (w/v) agar, 0.75% (w/v) sucrose] and placed at 4 °C for 2 days for vernalization, they then were placed in growth cabinets at 22 ± 1 °C for 7 days. Fifteen seedlings were transferred to Petri dishes containing 1× MS with 1.5% (w/v) sucrose, 1.4% (w/v) agar, the pH of the medium was adjusted to 7.0 and the seedlings were grown at 22 ± 1 °C for 16 days in a 16-h-light/8-h-dark cycle. After that period of time, *Trichoderma atroviride* was inoculated on the MS media as described below.

For Trichoderma distant interaction, pot interactions, ecotype Ms-0, and others microbe interactions, *A. thaliana* (Col-0 and Ms-0) seeds were surface-sterilized and sown on Petri dishes containing 0.2× MS medium [1% (w/v) agar, 0.75% (w/v) sucrose, the pH of the medium was adjusted to 7.0] and placed at 4 °C for 2 days for vernalization, they then were placed in growth cabinets at 22 ± 1 °C in a 16-h-light/8-h-dark cycle for 15–20 days. After that period of time, seedlings were inoculated with the respective culture of microbes on the MS media as described below.

### 4.2. Microorganism Growth Conditions and Seedling Inoculations

In a direct interaction experiment, *T. atroviride* (IMI206040) was grown on PDA plates for 8 days at 28 °C. Conidia were collected in distilled water and adjusted to a density of 1 × 10^3^ spores per mL. Then 10 μL of the spore suspension were applied on roots of 25-day-old *A. thaliana* plantlets grown in Petri dishes with MS medium and cultured for different periods: 48, 72, 96 and 120 h post-inoculation (hpi), control non-inoculated plants were cultured at the same times points. On the other hand, in a distant interaction experiment, 10 μL of a suspension of 1 × 10^6^ spores were applied at 3 cm of distance from the roots at the opposite ends of MS plates. Inoculated and non-inoculated plants were cultured for 48, 72, 96 and 120 h. After each treatment, plants were harvested and frozen in liquid nitrogen for RNA extraction. In soil experiments the Arabidopsis seedlings were sown in pots containing a soil mixture (one plant per pot). Each pot was maintained in a growth chamber (22 ± 1 °C) under a photoperiod of 16 h light/8 h dark until the plants were 1 month old. Then, three plants were inoculated with 1 mL of 1 × 10^6^ spore suspension of *T. atroviride* and 1 mL of water was added to three uninoculated control plants. Aerial parts were harvested and frozen in liquid nitrogen at 2, 4 and 6 dpi.

For *A. thaliana* Ms-0 interaction with *T. atroviride*, the 15-day-old plantlets were inoculated with 10 μL of the spore suspension (1 × 10^6^ spores) at the bottom of the plates close to the plantlets roots, inoculated, and control plantlets were harvested and frozen in liquid nitrogen at 48, 72, 96 and 120 h.

The plant pathogen *Pseudomonas syringae* pv. *tomato* DC3000 was cultivated on King’s B medium [50] with appropriate antibiotics at 28 °C, when bacterial culture reached mid to late log phase growth (OD_600_ = 0.6 to 1.0) the bacteria from liquid culture were harvested. Cells were washed once and resuspended in 10 mM MgCl_2_ solution, the OD_600_ was adjusted to 0.002 (1 × 10^6^ cfu/mL) and 10 μL of bacterial suspensions were added to each of the 20-day-old seedlings on the foliar area. Then the plantlets were harvest and frozen at different times (12, 24, 48 and 72 hpi) of interaction for RNA extraction.

The plant growth-promoting rhizobacterium *P. fluorescens* was isolated in our research group from sugar cane rhizosphere, the bacterium was grown on LB medium at 28 °C until the early stationary phase. Just before inoculation, cells were washed once and resuspended in 10 mM MgCl_2_ solution; the OD_600_ was adjusted to 0.002 (1 × 10^6^ cfu/mL), and 10 μL bacterial suspension were added to the roots of each of the 20-day-old plantlets. At different time points post-inoculation (72, 96 and 120 h) the plants were harvested and frozen; similarly, uninoculated control plants were harvest at the same times for RNA extraction.

### 4.3. Phytohormone Application

Phytohormones were applied as a spray to 15-day-old plantlets with 0.5× MS solution containing ethephon, SA and MeJA at the rate of 5 mM, 5 mM and 100 μM, respectively. The MS plates with treated seedlings were sealed and the plantlets were harvested at 1, 3 and 24 h after each treatment. Control plants were sprayed with 0.5× MS solution and sealed and harvest at the same time points as the treatment plants.

### 4.4. RNA Extraction and Real-Time qRT-PCR

Total RNA was extracted from inoculated and non-inoculated plants using the Concert™ Plant RNA reagent (Invitrogen, Carlsbad, CA, USA). Real-time PCR was performed in 10 μL of reaction mixture made up of 5 μL of Power SYBR^®^ Green RT-PCR Mix (2×), 200 nM of each oligonucleotide, 50 ng of RNA template and 0.08 μL of RT Enzyme Mix (125×) for one-step RT-PCR, using an StepOne Real-Time PCR Detection System and StepOne Software v2.1 (Applied Biosystems). The thermal cycling conditions consisted of 30 min at 48 °C (cDNA synthesis), 10 min at 95 °C (Activation of AmpliTaq Gold^®^ DNA polymerase), followed by 40 cycles for PCR cycling of 15 s at 95 °C for denature and anneal/extend of 1 min at 60 °C. These PCR reactions were repeated by triplicate for each condition. Quantification of *HR4*, *RPW8.1* and *RPW8.2* gene expression was based on a cycle threshold value and normalized to the *actin 8* (At1g49240) gene values. Absence of contaminant genomic DNA was confirmed by reactions in which no RT Enzyme Mix reverse transcriptase was added, and also primers for the *actin 8* gene were designed between two exons. The primers used were: 5′-CATCTCGAGAGACGAGAGCTAA-3′ and 5′-CTGAAGCCGTCGTAAATGACTT-3′ for *HR4* (At3g50480) transcript, 5′-GGACACTAAACTTGCTGAAGTTA-3′ and 5′-CAATAAT TATGGGGAATAAGAGAGA-3′ for *RPW8.1* transcript, 5′-ACAAAATAATGCCTCAACCGAAG-3′ and 5′-TGAGTCGTTTGACACAATTGGG-3′ for *RPW8.2* transcript and 5′-GCCAGTGGTCGT ACAACCG-3′ and 5′-TCATGAGGTAATCAGTAAGGTCAC-3′ for *actin 8*.

## 5. Conclusions

Whereas many *R* genes have been characterized in plant-pathogen interactions, such as *RPW8* genes, little is known about *R* genes involved in mutualistic interactions. Our results indicate that the *HR4* gene modulates their expression in interaction with the tested microbes. It was induced in particular in *Trichoderma atroviride* interaction, suggesting that it plays a role in the process of recognition and/or establishment of the interaction. When analyzing the Arabidopsis ecotype Ms-0, which has *RPW8* genes instead of *HR4*, it was observed that these genes are also responsive to *T. atroviride*, suggesting that this type of *R* genes is regulated in beneficial interactions.
